# Prospecting for Energy-Rich Renewable Raw Materials: *Agave* Leaf Case Study

**DOI:** 10.1371/journal.pone.0135382

**Published:** 2015-08-25

**Authors:** Kendall R. Corbin, Caitlin S. Byrt, Stefan Bauer, Seth DeBolt, Don Chambers, Joseph A. M. Holtum, Ghazwan Karem, Marilyn Henderson, Jelle Lahnstein, Cherie T. Beahan, Antony Bacic, Geoffrey B. Fincher, Natalie S. Betts, Rachel A. Burton

**Affiliations:** 1 The Australian Research Council Centre of Excellence in Plant Cell Walls, School of Agriculture, Food and Wine, University of Adelaide, Adelaide, South Australia, Australia; 2 Energy Biosciences Institute, University of California, Berkeley, California, United States of America; 3 Department of Horticulture, University of Kentucky, Lexington, Kentucky, United States of America; 4 AUSAGAVE, Aldgate, South Australia, Australia; 5 School of Marine and Tropical Biology, James Cook University, Townsville, Queensland, Australia; 6 The Australian Research Council Centre of Excellence in Plant Cell Walls, School of Botany, University of Melbourne, Melbourne, Victoria, Australia; Ohio University, UNITED STATES

## Abstract

Plant biomass from different species is heterogeneous, and this diversity in composition can be mined to identify materials of value to fuel and chemical industries. *Agave* produces high yields of energy-rich biomass, and the sugar-rich stem tissue has traditionally been used to make alcoholic beverages. Here, the compositions of *Agave americana* and *Agave tequilana* leaves are determined, particularly in the context of bioethanol production. *Agave* leaf cell wall polysaccharide content was characterized by linkage analysis, non-cellulosic polysaccharides such as pectins were observed by immuno-microscopy, and leaf juice composition was determined by liquid chromatography. *Agave* leaves are fruit-like—rich in moisture, soluble sugars and pectin. The dry leaf fiber was composed of crystalline cellulose (47–50% w/w) and non-cellulosic polysaccharides (16–22% w/w), and whole leaves were low in lignin (9–13% w/w). Of the dry mass of whole *Agave* leaves, 85–95% consisted of soluble sugars, cellulose, non-cellulosic polysaccharides, lignin, acetate, protein and minerals. Juice pressed from the *Agave* leaves accounted for 69% of the fresh weight and was rich in glucose and fructose. Hydrolysis of the fructan oligosaccharides doubled the amount of fermentable fructose in *A*. *tequilana* leaf juice samples and the concentration of fermentable hexose sugars was 41–48 g/L. In agricultural production systems such as the tequila making, *Agave* leaves are discarded as waste. Theoretically, up to 4000 L/ha/yr of bioethanol could be produced from juice extracted from waste *Agave* leaves. Using standard *Saccharomyces cerevisiae* strains to ferment *Agave* juice, we observed ethanol yields that were 66% of the theoretical yields. These data indicate that *Agave* could rival currently used bioethanol feedstocks, particularly if the fermentation organisms and conditions were adapted to suit *Agave* leaf composition.

## Introduction

Plant biomass is a source of chemical energy that can be converted to combustible transport fuels and biochemicals by fermentation or chemical conversion of plant-derived sugars [1]. Currently, plant materials from farming-intensive food production systems, such as corn, wheat grain or cane sugar, are being used to make bioethanol and biochemicals. In the future, alternative sources of energy-rich plant material from low-input systems that are independent from the food chain will be needed [2,3].

Plant biomass contains soluble and structural sugars: for example the vacuoles of storage cells in the stem of sugarcane contain high concentrations of sucrose, a soluble disaccharide and the cell walls in the trunks of willow trees contain a large amount of cellulose, a structural sugar composed of glucose [4]. The composition of historical agriculture plant species have been reported (Table 1; [5]); however, the relative importance of plant species is likely to change as agricultural industries adapt to new markets and climate change. Research into novel plants may reveal non-food sources of valuable raw materials. One example of a plant species that is likely to gain importance is *Agave*. Historically *Agave* has been used for production of alcoholic beverages, fibers, chemicals and sugar additives [6] and there is growing interest in using *Agave* for biofuel production.

**Table 1:** Comparison of potential biofuel feedstocks.

Species	Common name	Tissue	Cellulose (% w/w)	Non-cellulosic polysaccharides (% w/w)	Lignin (% w/w)
***Zea mays***	Corn	Stover without cobs	31–38	19–25	17–21
***Triticum aestivum***	Wheat	Whole plant	33	23	17
***Saccharum* spp.**	Sugarcane	Bagasse	32–43	12–26	23–28
***Sorghum bicolor***	Sorghum	Whole plant	23	14	11
***Panicum virgatum***	Switchgrass	Whole plant	30–35	24–28	17–20
***Populus* spp.**	Hybrid poplar	Whole tree without leaves	41–43	17–20	24–28
***Agave* spp.**	Agave	Whole residue from tequila brewing	31	17	17

Cellulose is the major source of glucose in feedstocks. Non-cellulosic polysaccharides contribute some fermentable hexose (glucose and galactose) and pentose (xylose and arabinose) sugars. Lignin is a non-sugar polymer that inhibits cell wall degradation and subsequent fermentation. Data are presented as percentage of dry weight (% w/w). Data may be accessed through the United States Department of Energy, Energy Efficiency & Renewable Energy, Biomass Feedstock Composition and Property Database, 2013 [5].

Alcoholic beverages such as tequila and mescal are made from the stem tissue of *A*. *tequilana* plants that are 8–12 years old. Fructans in mature stem tissue are degraded by heat to release fermentable fructose [7] and the leaves, which account for up to 66% dry weight of the biomass, are discarded [8]. *Agave* is a productive water-use efficient plant that grows in regions with extreme environments [9–11] and recent literature has considered the potential for using *Agave* as a feedstock for bioethanol production [12–18]. However, the composition of *Agave* leaf tissues from plants at an earlier stage in development has not been well characterized and may represent an energy-rich raw material that can be produced rapidly in a low-input system [19,20].

There are standard protocols for determining the composition of plant biomass, such as the analytical procedures published by the United States Government National Renewable Energy Laboratory (NREL) [21–26]. Biomass composition analyses may include determination of moisture content, total solids, acid-soluble and insoluble residues and the amount of water soluble carbohydrates (WSC), starch, mineral, lignin, protein, crystalline cellulose and non-cellulosic polysaccharides. In the context of using biomass to make biofuels and biochemicals, it is of interest to determine not only the amount of fermentable sugars that can be extracted from plant biomass, but also the amount of inhibitory compounds that are formed during processing which may interfere with conversion of the biomass to bioethanol [27]. For example, acetic acid is generated from the hydrolysis of acetyl groups associated with non-cellulosic polysaccharides. Weak acids like acetic can reduce yeast growth and ethanol yields by prohibiting monosaccharide metabolism and causing intracellular anion accumulation [27]. In addition, the compositions and proportions of sugar present in soluble forms and structural forms, and the recalcitrance of these structural sugars are important as they influence the processing methods and costs. These data are also used to estimate the bioethanol yields for a feedstock of interest.

Here, the composition of *Agave* leaves is determined, including a detailed analysis of the fermentable and non-fermentable compounds in *A*. *americana* and *A*. *tequilana*. The efficiency of enzymatic hydrolysis of *Agave* leaf cellulose and hydrolysis of fructans in juice samples is quantified. Compositional data is then extrapolated to calculate theoretical ethanol yields and *A*. *tequilana* leaf juice is fermented using two *Saccharomyces cerevisiae* strains. These compositional and fermentation data can be used to inform the development of biotechnology to exploit this energy-rich raw material.

## Material and Methods

### Plant material

*A*. *tequilana* and *A*. *americana* plants were approximately 2–3 y old at the time of harvest and had begun to reproduce asexually. The heights of the plants from the base to the tip of the tallest leaf were at least 2 m. Six plants of *A*. *tequilana* were harvested from Ayr (Queensland, Australia) and six plants of *A*. *americana* were harvested from the Adelaide Hills (South Australia, Australia). From each individual plant stem tissue and at least three leaves were collected. Permission for the described field studies were granted by either the crop manager or land owner.

**Fig 1:**
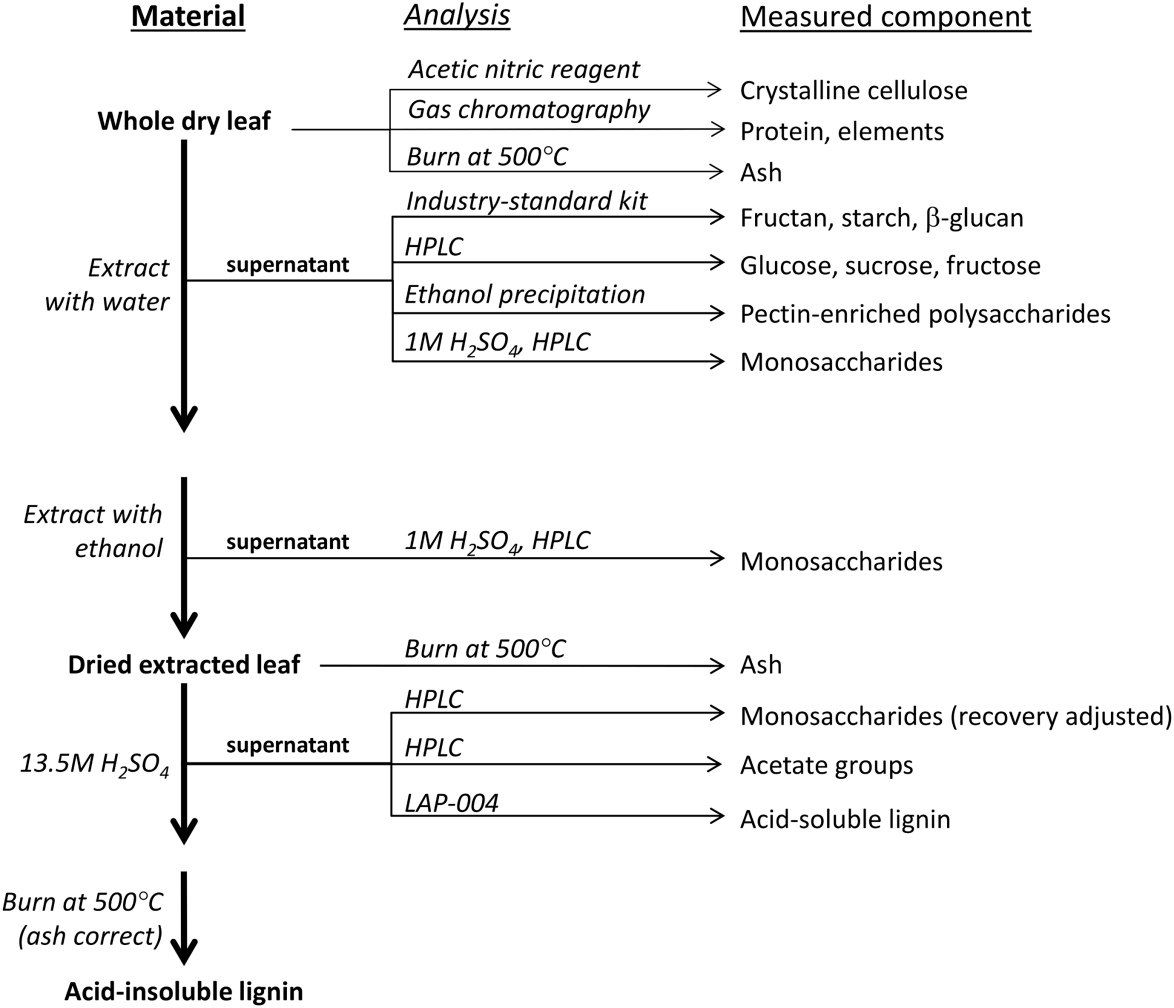
Flowchart outlining the steps taken to process and analyze *Agave* leaves.

The stem and leaves were separated at the time of harvest and fresh weights recorded. Juice from the stem tissue of each *A*. *tequilana* plant was collected after shredding (Cutter-Grinder CG03, Jeffco) and three leaves per plant (*A*. *americana* and *A*. *tequilana*) were collected for compositional analysis. A subset of the remaining leaves was pooled and two experimental shredders were used to extract juice (Cutter-Grinder CG03, Jeffco and Food processor, Abode). Wet bagasse was dried at 60°C to a constant moisture content. Juice and whole leaves were transported to the University of Adelaide on dry ice and stored at –80°C. Prior to analysis, samples were cut into 200–400 mm^2^ pieces, weighed, lyophilized (Labconco-Freezone, Missouri, United States) and moisture loss was calculated. Dried leaf material was ground in a 25 mL stainless steel grinding jar with one 7 mm steel ball. The grinding jars were shaken at 30 Hz for 3 min (Retsch mill MM400, Retsch GmbH; Haan, Germany). A flowchart of methods employed for compositional analysis is included in Fig 1.

#### Fiber extraction

Whole leaves were frozen at –80°C and subsequently thawed at room temperature. Fibers were pulled from three plants of each species and separated from the vegetative tissue manually. The fibers were further cleaned using forceps to remove any attached pith tissue. Fibers (1–2 mm) were dried overnight at 60°C. Dried fibers were hydrolyzed using 1M sulfuric acid (H_2_SO_4_) for 3 h at 100°C [28], cooled and centrifuged at 28 000 g for 5 min. The monosaccharides in the supernatant were analyzed using high-performance liquid chromatography (HPLC). Derivatisation and quantification of monosaccharides was completed according to [29] with modifications to the gradient conditions. Elution was performed with 10% acetonitrile, 40mM ammonium acetate (A) and 70% acetonitrile (B) at a flow rate of 0.8 mL/min. The gradient for solvent B is as follows: 0–9.5 min, 8% B; 9.5–10 min, 17% B; 10–11.5 min, 100% B; 11.5–14.5 min, 8% B.

### Measurement of leaf composition

#### Total soluble solids (TSS) in *Agave* juice

Aluminum pans (Fisher Scientific, Australia) were dried at 60°C and their initial weight recorded. Juice samples were centrifuged at 10 000 g for 10 min and 2 mL aliquots of supernatant were added to the pans and heated at 60°C for 48 h, leaving a solid residue in the pan. The final weight of the pan and solid residue was subtracted from the initial weight to calculate the total soluble solids (TSS).

#### Crystalline cellulose

Crystalline cellulose in leaf tissue and fiber-enriched samples was determined using a modified Updegraff method according to [30].

#### Elemental analysis and protein and mineral (ash) quantification

Samples for the elemental analysis included 300 mg of dry, ball milled, whole leaf tissue or 1 mL of juice. Elements (Al, Ca, Fe, Mg, P, K, Na, S and Zn) were measured using a closed tube nitric acid/hydrogen peroxide digest and radial view inductively couple plasma-optical emission spectrometry [31].

The total nitrogen content was measured by the Waite Analytical Services, University of Adelaide using complete combustion gas chromatography (Carlo Erba Instrument) and 100 mg of biomass or 1 mL of juice. The nitrogen value was converted to an estimate of the protein content using the nitrogen factor (NF) 6.25 [21]. Mineral content of extracted and non-extracted material was calculated by heating samples to 500°C for 3 h [22].

#### Water- and ethanol-soluble carbohydrates in *Agave* leaves

Leaf samples were dried at 60°C and extracted sequentially in water, 95% v/v ethanol and 70% v/v ethanol at 80°C for 15 min using a 1:5 ratio of biomass to extraction liquid. The residual biomass was dried at 60°C.

The total fructan and (1,3;1,4)-β-glucan content in water extracts was measured using commercial assay kits (Fructan HK-Megazyme: AOAC Method 999.03 and AACC Method 32.32 and AACC Method 76.13, Mixed-Linkage Beta-Glucan-Megazyme: AACC Method 32–23, AOAC Method 995.16, EBC Methods 3.11.1, 4.16.1, 8.11.1 and ICC Standard Method No. 166; International Ireland Ltd., Wicklow, Ireland), respectively.

Glucose, fructose and sucrose in water extracts were measured by hydrophilic interaction chromatography (HILIC), using a Prevail Carbohydrate ES column (150 × 4.6 mm) (Alltech; Illinois, United States) on an Agilent 1200 series liquid chromatography instrument equipped with an evaporative light scattering detector (Alltech ELSD 800). The mobile phase consisted of water (A) and 90% acetonitrile (B) at a flow rate of 1.0 mL/min at 20°C. The gradient for solvent B is as follows: 0–18 min, 94.5% B; 18–19 min, 64.5% B; 19–20 min, 0% B; 20–30 min; 94.5% B. The pectin-enriched polysaccharide content in water extracts was determined using an ethanol precipitation method according to [32].

Solvent was removed from water and ethanol extracts separately by centrifugal evaporation (Savant SC110 Speed Vac, Thermofisher; Massachusetts, United States). The concentrated material was hydrolyzed using 1M sulfuric acid (H_2_SO_4_) for monosaccharide analysis using HPLC, as previously described [29].

#### Measurement of structural carbohydrates, lignin and acetyl content

For compositional analysis, samples were extracted using an Automated Extraction System (ASE) following [23]. *Agave* leaves (cut to 2−4 mm in size); aluminum pans and Whatman GF/C 55 mm glass microfiber filters (Sigma-Aldrich, United States) were dried at 105°C. Extraction cells (11 mL) were fitted with pre-weighed filter paper and 1 g of dried material added. Material was extracted with three water cycles followed by three 190 proof ethanol cycles at 100°C (ASE300, Dionex). Extraction settings were modified to 60 s nitrogen purges following extraction, 5 min static time and 120% rinse volume. Following extraction the remaining alcohol insoluble residue (AIR) and filter paper were placed in pre-weighed aluminum pans and dried at 105°C. Dried, extracted biomass was ground using a Retsch mill MM400, as previously described. The percentage of extractables was calculated based on the difference between the initial weight (before water and ethanol extraction) and final weight (after extraction).

Following extraction the alcohol insoluble residue was analyzed following [24]. Briefly, a 30 mg sample of dried ground material was treated with 13.5M sulfuric acid at room temperature for 1 h. The samples were diluted to 0.75M acid and autoclaved at 121°C for 15 minand centrifuged for 10 min at 10 000 g. The supernatant was collected for monosaccharide, acid-soluble lignin and acetate analyses. A sugar recovery standard for monosaccharides was carried through the acid hydrolysis as outlined in [25]. Monosaccharides were measured following derivatisation as previously described using HPLC. The acid-soluble lignin content was measured using a spectrophotometer (Thermo Fischer, Waltham, MA, USA) set at a wavelength of 205nm and calculated following LAP-004 using the extinction coefficient value 110 L/g-cm [26]. The acetyl content in the supernatant was analyzed at 60°C using an Aminex HPX-87H column (300 x 7.8 mm) (Bio Rad; California, United States) on a 1100 series liquid chromatography instrument. Elution was performed isocratically with 2.5mM H_2_SO_4_ at a rate of 0.5 mL/min [33]. Starch was measured in extracted samples following a commercial assay (Total Starch-Megazyme: AOAC Method 996.11; International Ireland Ltd., Wicklow, Ireland).

The residual biomass was washed to a neutral pH and filtered through pre-dried and pre-weighed Whatman GF/C 55 mm glass microfiber filters (Sigma-Aldrich, United States). The filter paper and collected sample residue was heated to 105°C overnight and weighed (*M1*). The material was ash corrected by heating at 500°C for 3 h and weighed (*M2*). The lignin content was calculated based on the difference between *M2* –*M1* divided by the initial weight.

#### Linkage analysis of cell wall residue in whole leaf

Lyophilized leaf material was ground in a 25 mL stainless steel grinding jar with one 7 mm steel ball. The grinding jars were shaken at 30 Hz for 3 min (Retsch mill MM400, Retsch GmbH; Haan, Germany) until all cells were ruptured. Samples were extracted sequentially with 80% v/v ethanol on ice, and acetone and methanol at room temperature. Samples were digested with α-amylase (*B*. *licheniformis*; EC 3.2.1.1) to remove starch. Linkage analysis and carboxyl reduction of the material followed [34].

### Enzymatic saccharification

For saccharification, Celluclast 1.5 L (cellulase preparation from *Trichoderma reesei*) and Novozyme 188 (cellobiase preparation from *Aspergillus niger*) (Sigma-Aldrich; St Louis, MO, USA) were mixed in equal volumes. Enzymatic activity of the cellulase cocktail was measured according to the National Renewable Energy Laboratory (NREL) analytical procedure, Measurement of Cellulase Activities (LAP 006) [35]. The saccharifications used an enzyme concentration of 60 filter paper units (FPU). Alcohol insoluble cell walls were prepared according to [36]. Modifications to the micro scale saccharification were made using equivalent amounts of 0.02 g cellulose for all samples (NREL; LAP 009) and the total reaction volume reduced to 1.5 mL [37,38]. The glucose concentration was measured using a Yellow Springs Instrument (YSI) glucose analyzer (Yellow Springs, OH, USA) over 48 h, n = 3.

### Analysis of hydrolyzed juice fraction

Samples of diluted, centrifuged, juice were treated with trifluoroacetic acid (TFA) to a final concentration of 0.2M TFA or fructanase (Fructan HK-Megazyme: AOAC Method 999.03; International Ireland Ltd., Wicklow, Ireland). For the TFA hydrolysis, juice and acid were mixed in equal proportions and samples were heated at 80°C for 1 h. For enzymatic hydrolysis, juice and enzyme mix were combined in equal proportions and samples incubated at room temperature for 30 min, then heated to 100°C for 15 min to deactivate the enzyme. Carbohydrates in the raw and treated juice samples were measured by HILIC, using a Prevail Carbohydrate ES column (150 × 4.6 mm) as previously described.

### Microscopy

Fresh tissue was fixed in a solution of 0.25% glutaraldehyde, 4% paraformaldehyde and 4% sucrose in phosphate-buffered saline (PBS) for 24 h at 20°C. Samples were washed twice with PBS, dehydrated in an ethanol series, infiltrated in LR White resin (ProSciTech Pty Ltd, Australia), and polymerized in a gelatin capsules at 58°C for 48 h [39,40].

#### Light microscopy

Embedded *Agave* leaf tissue was sectioned at 1 μm using a diamond knife on a Leica Ultracut R microtome. Sections were collected and dried onto poly-L-Lysine-coated microscope slides and stained with either toluidine blue (Sigma-Aldrich, United States) or methylene blue/basic fuchsin (ProSciTech Pty Ltd, Australia). Sections were viewed using a Leica light microscope (Version 4.3) and images captured with a Zeiss M2 Axio Imager fitted with an MRm Rev. 3 AxioCam.

#### Immuno-electron microscopy

Ultrathin sections of 70–90 nm were collected on collodion-coated nickel grids and labeled following Aurion Immunogold Specific Localisation Methods [41] using the primary antibodies LM19 (diluted 1/20), LM11 (diluted 1/500), LM20 (diluted 1/20) (Plant Probes, UK), or (1→4)-β-Mannan (diluted 1/50; Biosupplies, AU) [42–44]. Diluted (1/30) secondary antibodies goat-anti-rat IgM (LM19, LM11 and LM20; Jackson ImmunoResearch Labs Inc., USA) and goat-anti-mouse IgG (Mannan; ProSciTech, Australia) were used. Labeled sections were examined and imaged using a Philips CM100 Transmission Electron Microscope.

### Preparation of inoculums, fermentation conditions and analysis

Two *Saccharomyces cerevisiae* strains (Y-139 and Y-636) were kindly provided by the ARS Culture (NRRL) Collection, National Center for Agricultural Utilization Research (Peoria, IL, USA). Strains were streaked on 1% w/v yeast extract, 2% w/v peptone, 2% w/v glucose and 2% w/v agar (YPD) plates. Plates were grown overnight at 28°C and a single colony picked. The single colony was grown in YPD liquid broth (28°C) in a shaker incubator (120 rpm). The YPD cultures were used to inoculate autoclaved *Agave* leaf juice at a cell density of 5 x 10^6^ cells/mL. Juice samples were autoclaved (121°C, 15 min) and centrifuged at 5000 rpm for 10 min to remove excess leaf tissue. The fermentations were completed in Erlenmeyer flasks with side arm sampling ports and sealed with water-filled airlocks. The fermentation flasks were placed in a shaker (150 rpm) set at 28°C for 96 h. The cells were removed from the fermentation broth by centrifugation (1m / 10 000 g) and the supernatant stored at -20°C until analysis. Ethanol concentration was determined using an Aminex HPX-87H column (300 x 7.8 mm) (Bio Rad; California, United States) as described above, following [33].

## Results and Discussion

### Processing of *Agave* biomass: leaf and stem fractions

**Fig 2:**
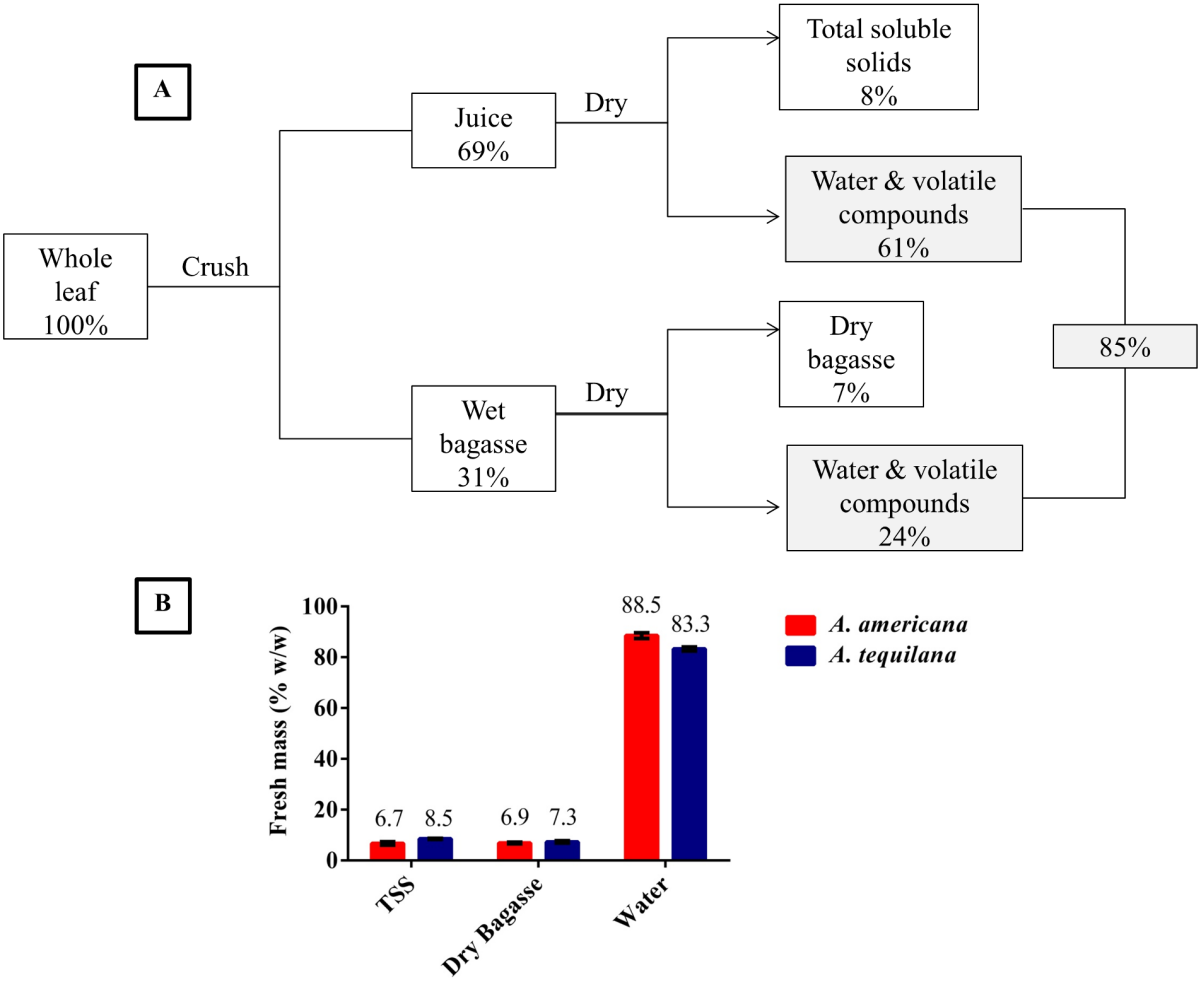
*Agave* processing and moisture content. Whole leaves were crushed, producing juice and wet bagasse fractions (a). These fractions were dried separately to calculate moisture content. Data is presented as percentage of fresh (wet) starting mass (% w/w). The values shown in gray are used to calculate total moisture content. The distribution of leaf fresh mass (% w/w) in *A*. *americana* and *A*. *tequilana* (b).

One feature of *Agave* plants that differs from traditional biofuel feedstocks is its high moisture content and inversely, its low water requirements. The seasonal water requirement of *Agave* (300–800 mm/yr) is minor compared with other biomass sources such as sugarcane (*Saccharum* spp., 1500–2500 mm/yr) [18]. The lower water requirement for *Agave* is attributed to its ability to store large volumes of water in its leaves (>83% w/w) (Fig 2). This water storage is common for crassulacean acid metabolism (CAM) plant assimilatory organs and aids in buffering the plant against periods of extended drought [45]. Such physiological characteristics make *Agave* a favorable biofuel feedstock for dry, marginal regions. However, moisture content directly contributes to biomass weight, which affects transport and processing costs. Separating *Agave* juice from the biomass at the time of harvest may result in higher yields and lower input costs such as transportation.

**Fig 3:**
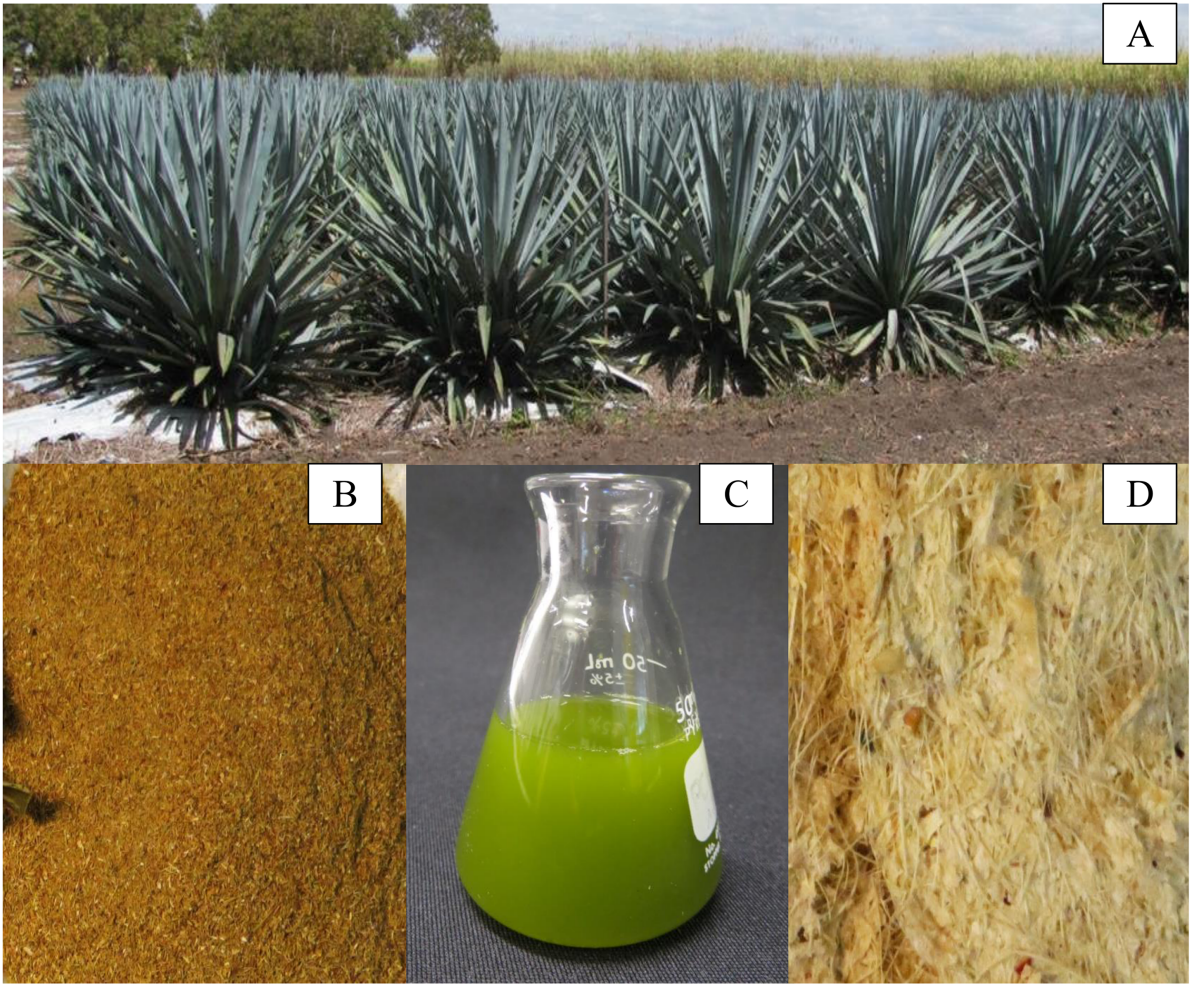
Different fractions of *Agave* material. Two year old *A*. *tequilana* plants in Australia (a). Partially dried leaves reduced to smaller particle sizes using a ball mill (b). Juice extracted from leaves using an experimental shredder (c). Dried fibers after extraction from wet bagasse (d).

The above-ground portion of *Agave* plants can be separated into leaves and stems (Fig 3a). For 3 year old *Agave* plants, the ratio of leaf: stem dry weight is 4:1, but becomes more variable with age [8]. Whole leaf and stem tissue may be dried and ground to remove excess moisture and to reduce particle size (Fig 3b). Alternatively, crushing the leaves by mechanical force releases 69% of the wet weight (Fig 2a) as a sugar-rich juice (Fig 3c). The biomass that remains after crushing is a fibrous bagasse, which may be further dried to remove excess moisture (Fig 3d).

### Analysis of the whole leaf fraction

#### Pectic polysaccharides occur in crystal sheaths

**Fig 4:**
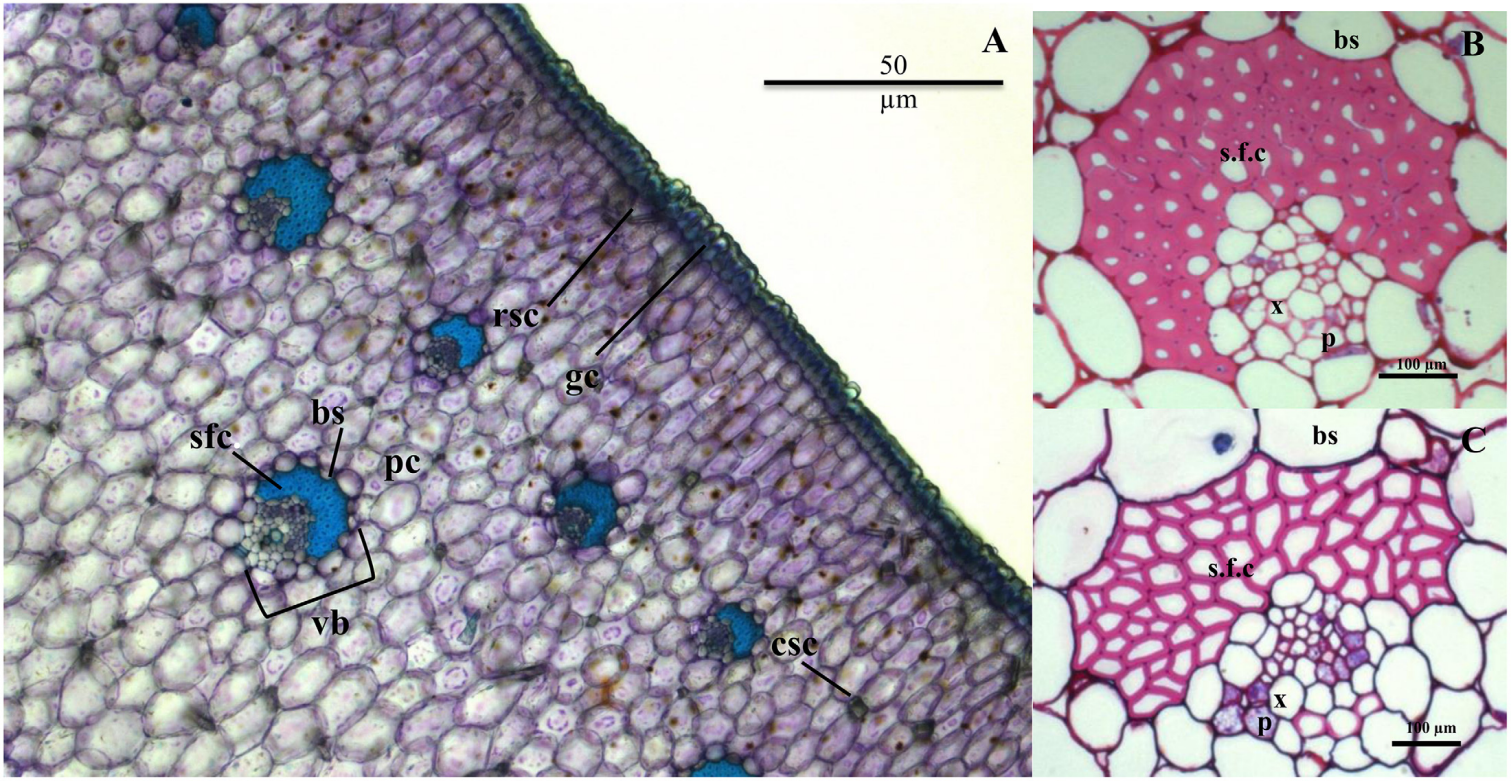
*Agave* leaf morphology. Transverse section of *A*. *tequilana* leaf stained with toluidine blue (a). Crystals are situated at the junction between some parenchyma cells within the tissue and at the site of stomata at the epidermis. Vascular bundles and fibers in *A*. *tequilana* (b) and *A*. *americana* leaf (c) stained with basic fuchsin. Sclerenchymatous fiber cap (sfc); bundle sheath (bs); parenchyma cells (pc); guard cells (gc); cubic shaped crystals (csc); rod shaped crystals (rsc); vascular bundle (vb)

The morphology of *Agave* cells and the spatial localization of polysaccharides in the leaf tissue was investigated. Transverse sections of *A*. *tequiliana* leaf were stained with toluidine blue to observe the morphology of the tissues (Fig 4a). Toluidine blue recognizes carboxyl groups on polysaccharides and proteins, and shows the distribution, but not amount or structure, of polysaccharides. Staining was observed in and around the parenchyma cells, with sclerenchymatous fiber cap cells staining very brightly. Further examination revealed that the sclerenchymatous fiber caps around the vascular bundles in *A*. *tequilana* (Fig 4b) had thicker cell walls than in *A*. *americana* (Fig 4c). These fiber caps surrounding the xylem and phloem cells are the main structural support for the leaves [46], and the thicker cell walls explain the more erect leaf morphology of *A*. *tequilana* plants.

**Fig 5:**
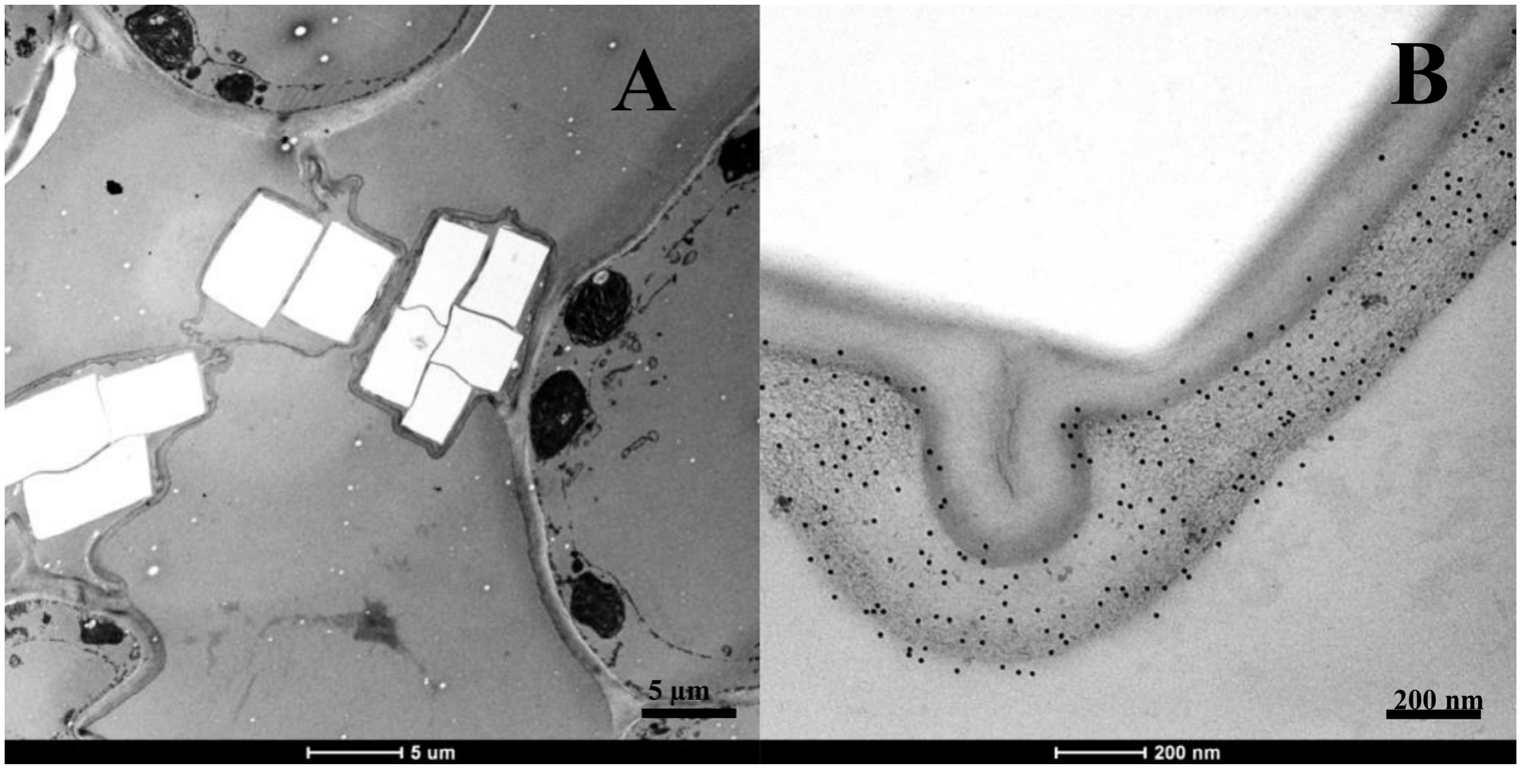
*Agave* tissue has pectinaceous crystal clusters localized at cell junctions. Transmission electron microscopy (TEM) image of crystals between junctions of cells (a) in *A*. *tequilana*. Labeling of methyl-esterified homogalacturonan (pectin) with LM20, was identified in the outer sheath of the crystals (b).

Crystal clusters were identified at the junction between cells in *Agave* leaf tissue (Fig 5a). Crystals have been identified in a range of photosynthetic organisms but the abundance, distribution and crystal structure varies between organisms and within tissue types [47]. The accumulation of crystals is correlated with oxalic acid production in plant tissue during normal development and in fungal-plant symbiosis [48]. A pectin-specific antibody that detects methyl-esterified homogalacturonan (LM20) [44] revealed the presence of pectic polysaccharides in the sheath surrounding the crystals (Fig 5b). There is conflicting information about the sheath surrounding the crystals in *Agave* plants; our results support a finding that polysaccharides are present [49], but this is not consistent with another report indicating that no polysaccharides are present in this sheath [50].

**Fig 6:**
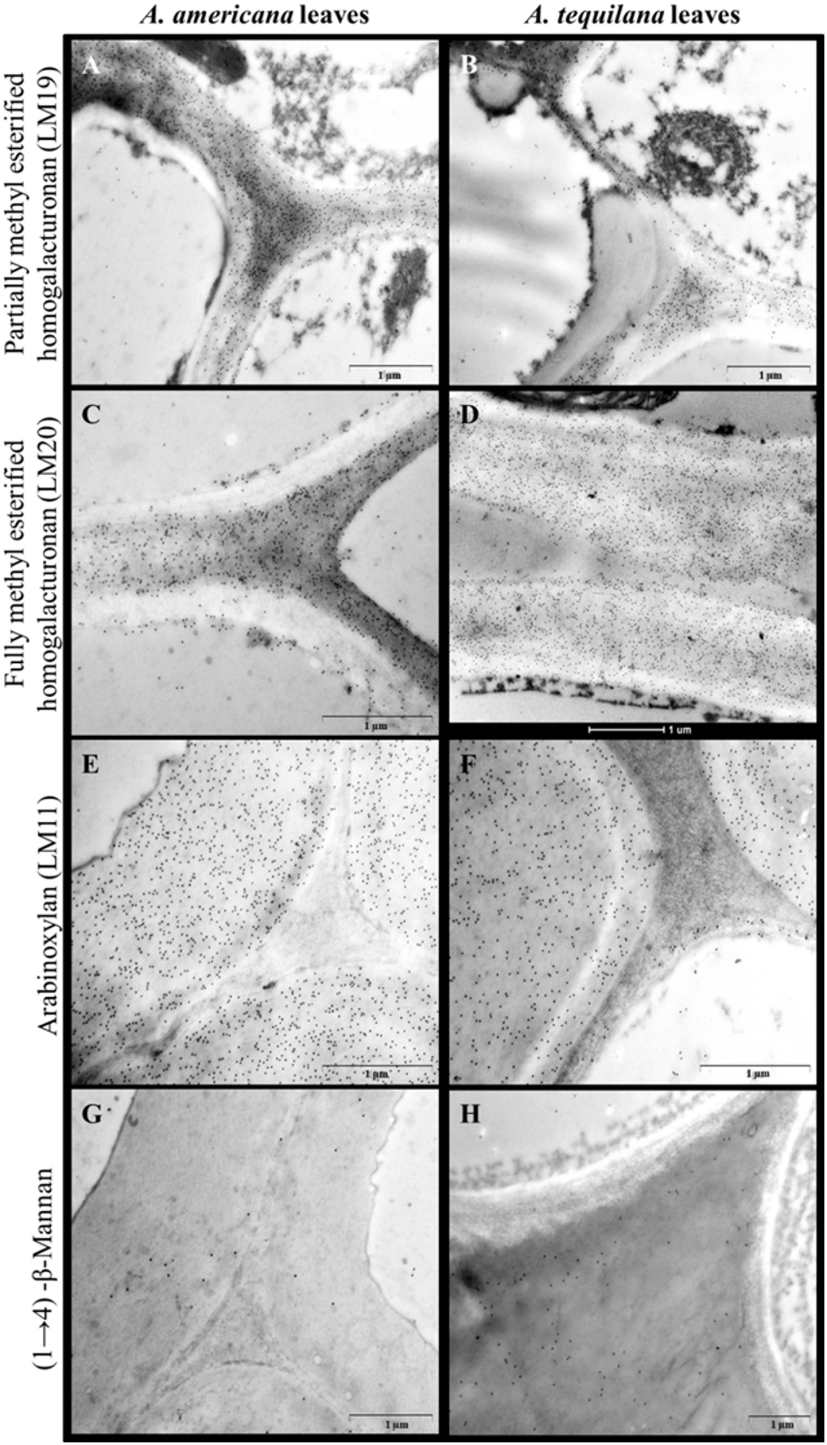
Cell wall polysaccharides detected by immunolabeling and transmission electron microscopy. Xylem tissue labeled with LM19, an antibody for partially methyl-esterified homogalacturonan (a-b) (pectin, [44]). Parenchyma cells labeled with LM20, an antibody for methyl-esterified homogalacturonans (c-d) [44]. Phloem tissue labeled with LM11 indicating the presence of arabinoxylan [42] (e-f). Leaf inner epidermal cells labeled with an antibody for (1→4)-β-mannan indicating the presence of mannan (g-h) [43]. Scale bars = 1μm.

Labeling of partially (LM19; [44]) and fully (LM20; [44]) methyl-esterified homogalacturonan was also observed in xylem parenchyma cell walls in both species (Fig 6a–6d)]. Both linkage analysis and results from the water soluble fraction confirm that high levels of pectins are present in *Agave* leaves. However, the amount of pectin-enriched polysaccharides in water extracts of *A*. *tequilana* was five times higher than in *A*. *americana* (Table 2); whereas linkage analysis indicated that homogalacturonan levels were considerably higher in *A*. *americana* (17.6 mol%) than in *A*. *tequilana* (6.5 mol%; Table 3). These data indicate that pectins in *A*. *tequilana* leaves may be more soluble than those in *A*. *americana*.

**Table 2:** Composition of *A*. *americana* and *A*. *tequilana* leaves.

	*A*. *americana* (% w/w)	*A*. *tequilana* (% w/w)
**Soluble extracts**	**55.5 ± 2.9**	**45.8 ± 2.5**
*WSC	9.1 ± 5.9	15.3 ± 3.0
Glucose	13.5 ± 3.6	4.6 ± 0.8
Fructose	7.8 ± 1.4	2.8 ± 0.6
Fructan	3.4 ± 2.5	4.9 ± 2.5
Sucrose	4.4 ± 0.5	3.0 ± 1.1
*Polysaccharides	4.0 ± 0.2	12.6 ± 1.1
Hydrolyzed monosaccharides	2.2 ± 0.3	2.4 ± 0.2
Ethanol-insoluble (pectin-enriched)	1.8 ± 0.4	10.2 ± 1.1
*Ethanol-soluble monosaccharides	6.0 ± 1.6	1.3 ± 0.2
Ash (non-structural inorganics)	*6*.*4 ± 1*.*4*	*15*.*1 ± 1*.*6*
*Other*	*10*.*0*	*1*.*5*
**Insoluble components**	**44.5 ± 2.9**	**54.1 ± 2.5**
*Monosaccharides	21.3 ± 1.7	26.1 ± 3.6
Glucose	12.0 ± 1.8	16.4 ± 2.3
^Starch	5.7 ± 1.4	1.4 ± 0.3
Xylose	2.9 ± 0.7	4.4 ± 0.7
Galacturonic acid	2.8 ± 0.2	3.1 ± 0.7
Galactose	2.7 ± 0.6	1.4 ± 0.1
Arabinose	0.9 ± 0.1	0.8 ± 0.1
Lignin	9.3 ± 0.9	12.7 ± 1.1
Acid-insoluble	5.3 ± 1.0	9.1 ± 1.4
Acid-soluble	4.0 ± 0.7	3.6 ± 0.3
Protein	6.2 ± 2.0	5.8 ± 0.7
Acetate groups	1.0 ± 0.2	0.7 ± 0.2
Ash (structural inorganics)	2.1 ± 1.0	5.5 ± 1.1
*Other*	*4*.*6*	*3*.*3*

The soluble extracts and insoluble residue, comprising structural carbohydrates and other cell wall components, were quantified (n = 6). Data are presented as percentage of dry weight (% w/w).

* indicates the values used to calculate total sugar content: 60.4% w/w for *A*. *americana* and 55.3% w/w for *A*. *tequilana*. Italics indicate values derived from calculation rather than direct measurement.

^Indicates values (starch) which were not included in the mass balance. Components of ‘Other’ (otherwise unaccounted for mass) are likely to be lipids and waxes in the soluble fraction or unhydrolyzed crystalline cellulose and pectin in the insoluble fraction.

**Table 3:** Polysaccharides detected by linkage analysis in *Agave* leaf.

Polysaccharide	*A*. *americana* (mol%)	*A*. *tequilana* (mol%)
Arabinan	5.5	4.7
Type I arabinogalactan	7.4	2.3
Type II arabinogalactan	2.4	1.5
Arabinoxylan	13.4	16.4
Cellulose	31.9	45.3
Heteromannan	6.6	6.0
Homogalacturonan	17.6	6.5
Rhamnogalactan I/II	0.7	0.3
Xyloglucan	10.6	12.7
Unassigned	3.9	4.3
**Total**	**100.0**	**100.0**

Polysaccharides detected in alcohol-insoluble residues (AIR) of *A*. *americana* and *A*. *tequilana* leaves (n = 3). Data are presented as relative percent molarity (mol%). Individual linkages were classified as described in S1 Table. Unassigned linkages include the linkages measured where the polysaccharide of origin was not clear.

The distribution of other cell wall polysaccharides was investigated using antibodies specific to xylan (LM11) [42] and (1→4)-β-mannan [43]. Xylan labeling was observed in the phloem walls (Fig 6e and 6f), consistent with linkage data (Table 3) indicating that heteroxylan is present in *Agave* cell walls. Mannan was detected to a similar extent in cell walls of parenchyma and inner epidermal tissue in both species (Fig 6g and 6h), again consistent with the linkage data (Table 3) that indicated heteromannan in both species.

#### The soluble fraction contains high levels of fermentable sugars

Sections of whole *Agave* leaves were dried, milled into fine particles, and sequentially extracted with water and ethanol to generate soluble and insoluble fractions. The water soluble carbohydrates (WSC), comprising glucose, fructose, fructans and sucrose, ranged from 15–29% dry weight. In mature *Agave* plants, fructans are the main storage carbohydrate in the stems [10]. Fructans were also the predominant WSC found in *A*. *tequilana* leaves, but *A*. *americana* leaves were richer in glucose, fructose and sucrose (Table 2). Total leaf WSC content was lower than the 36–64% w/w found in 6 year old *Agave* stems [10], which have been traditionally selected and used for tequila production, but was much higher than the 5% and 11% w/w found in the biofuel feedstock switchgrass (*Panicum virgatum*) [51] and fructan-rich chicory (*Cichorium intybus*) [52], respectively.

Other soluble sugars were analyzed by hydrolyzing acid-labile polysaccharides into monosaccharides, which were subsequently identified by HPLC. For both species, these monosaccharides comprised a very small proportion of the total mass (Table 2), which is not surprising as the higher molecular weight polymers usually have limited solubility in aqueous solutions [53]. Unhydrolyzed polysaccharides were precipitated with ethanol to create a pectin-enriched fraction [32], which, in *A*. *tequilana*, comprised over 10% of the dry weight of the leaves (Table 2). From a biofuel perspective, pectins play mixed roles: soluble pectins can be hydrolyzed into monosaccharides for fermentation [54], however acetate substituents on pectins can hinder hydrolysis by blocking cleavage sites for lytic enzymes [55] and once liberated from the polymer these compounds can be toxic to susceptible fermenting microorganisms such as *Pichia stipitis* [56]. Alternatively, when thermochemical conversion processes such as catalytic pyrolysis are used instead of fermentation to produce a hydrocarbon based biofuel the amount of non-carbohydrate cell wall components (i.e. acetyl) in the biomass is less important [57].

#### The insoluble fraction is predominantly cellulose with low levels of lignin

The remaining insoluble residue, largely cell wall material, was dried, milled, and hydrolyzed with concentrated sulfuric acid. The resulting monosaccharide profiles of *A*. *americana* and *A*. *tequilana* leaves were similar, with 12–16% w/w glucose, 3–4% w/w xylose, 3–4% w/w galacturonic acid, 1–3% w/w galactose and less than 1% w/w arabinose (Table 2). However, acid hydrolysis does not permit identification of cell wall polysaccharides, so linkage analysis was used to obtain structural information. Linkages were assigned to polysaccharides according to Pettolino *et al*., 2012 [34] (S1 Table).

For both species, the majority of the material was composed of hexose (C6) sugars. Cellulose was the most abundant polysaccharide, comprising 32–45 mol% of the cell walls (Table 3). *A*. *americana* leaf cell walls had higher amounts of pectin-associated polysaccharides such as Type I arabinogalactan and homogalacturonan. There was more heteroxylan in *A*. *tequilana* than in *A*. *americana* but the heteroxylan in *A*. *americana* was less substituted than the heteroxylan in *A*. *tequilana* (S1 Table). Xylans with low degrees of substitution are reported to bind more strongly to cellulose [58]. The amounts of other cell wall polysaccharides were similar between the two species (Table 3).

Starch, a (1,4)-α-glucan, was removed from the biomass samples prior to linkage analysis to reduce interference with cellulose quantification. Starch was measured separately using a commercial assay at 1−6% w/w (Table 2). The polysaccharide (1,3;1,4)-β-glucan was not detected by enzymatic assays or by linkage analysis.

The total lignin content of the leaves was 9.3–12.7% w/w (Table 2). Compared with other biofuel feedstock crops such as corn, sugarcane and poplar, which all have lignin contents >17% w/w (Table 2), *Agave* is considered a low lignin feedstock. Lignin is a non-sugar aromatic polymer that binds strongly to cell wall polysaccharides via covalent and non-covalent linkages. This barrier limits enzyme binding sites on the polymers and reduces the rate and efficiency of hydrolysis [59]. Alternatively, lignin can be acid-soluble. High levels of soluble lignin in the hydrolyzate can be an inhibitor to both yeast and bacteria, reducing the yield of ethanol produced [60]. In *Agave*, 28–43% of the total lignin was acid-soluble (Table 2). Acid-soluble lignin has been shown to be predominantly composed of syringyl lignin and, to a lesser degree, secondary hydrophilic compounds [61].

#### Cellulose undergoes 40% saccharification without pre-treatments

The predominant polysaccharide identified in both species of *Agave* using linkage analysis was cellulose (Table 3). Due to its recalcitrance, cellulose quantification after hydrolysis with sulphuric acid can be an underestimate [62]. As a result, a method optimized for the isolation and measurement of cellulose was employed [30]. The amount of cellulose in whole tissue was slightly lower in *A*. *americana* (15.7% w/w) than in *A*. *tequilana *(16.5% w/w).

**Fig 7:**
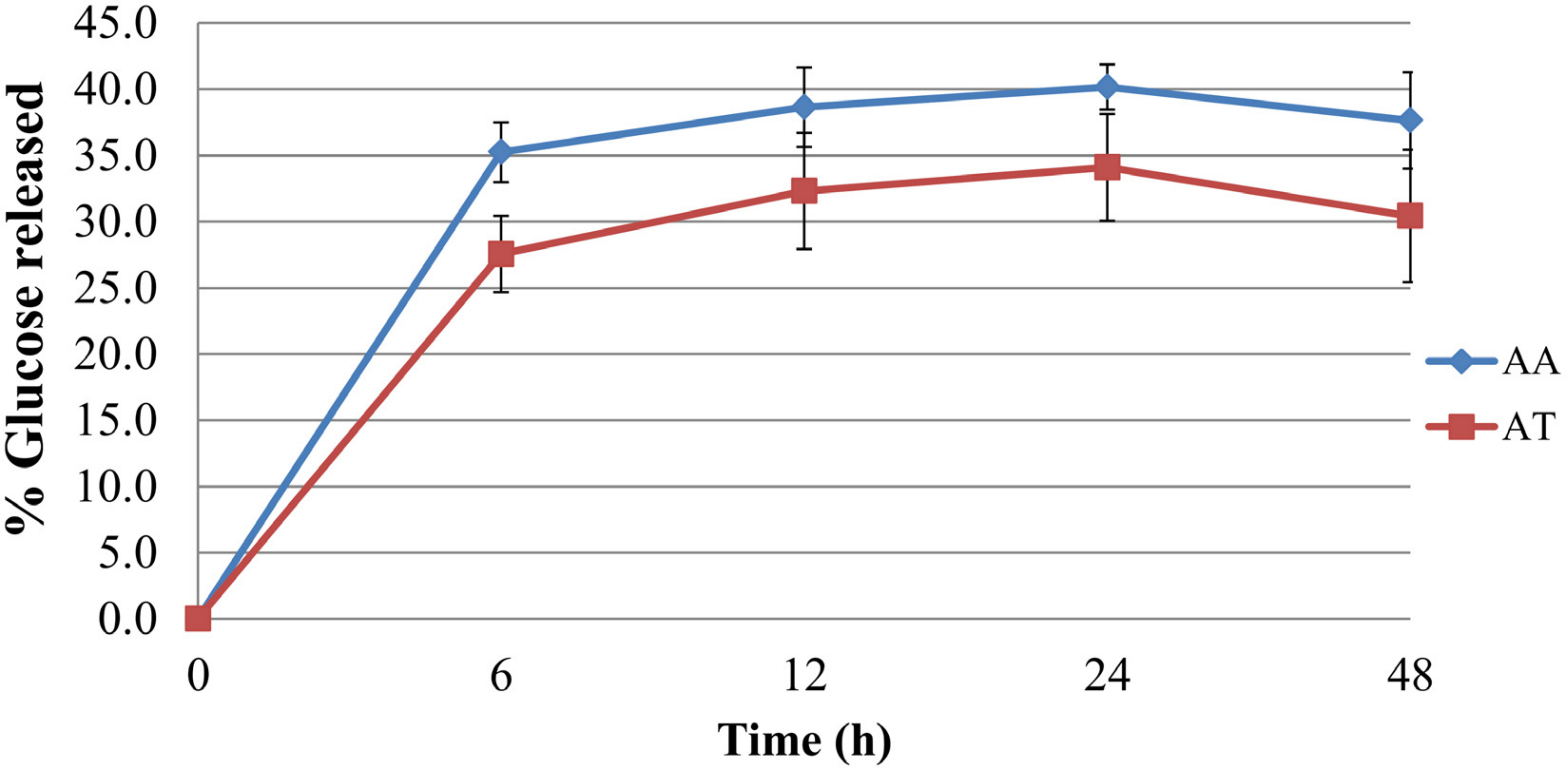
Cellulose, the most predominant polymer in *Agave* leaf tissue is degraded by cellulases. Liberation of the monomer glucose from the alcohol insoluble residue of *A*. *americana* (AA) and *A*. *tequilana* (AT) was measured over 48 h. The rate of saccharification is expressed as a percentage of cellulose converted into glucose (n = 3).

Cellulose is embedded *in muro* within a complex matrix of non-cellulosic polysaccharides, lignin and proteins. Saccharification tests were thus performed on the heterogeneous alcohol insoluble residue (removing all free glucose from the matrix) on identical cellulose loadings rather than on purified cellulose. The liberation of glucose was monitored over 48 h of enzymatic digestion using a cellulase cocktail. The extent of saccharification was similar for both species (40–35%) but slightly higher for *A*. *americana* (Fig 7). The efficiency of cellulose breakdown and therefore the total ethanol yield from *Agave* may be increased if the biomass is further processed using pre-treatments, thus loosening the bonds within and between cellulose chains.

### Analysis of leaf juice and fiber fractions

#### Agave leaf juice is rich in fructans

**Fig 8:**
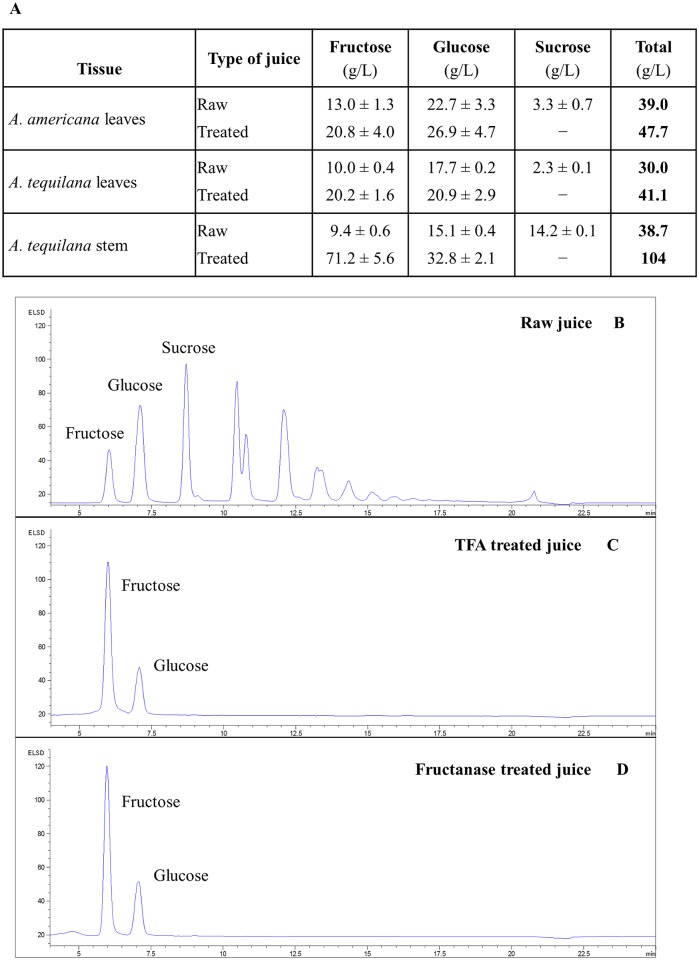
Quantification of juice sugars from *A*. *americana* leaves and *A*. *tequilana* leaves and stem. The amount of glucose, fructose and sucrose present in both raw and TFA-treated juice samples (a). Data are presented as g/L. Additional peaks for which there are no known standards were detected in the chromatograms of raw juice (b). *A*. *tequilana* stem juice is used as a representative of all three, very similar, chromatograms for the raw and treated samples. Chromatogram of TFA-treated *A*. *tequilana* stem juice (c). Chromatogram of fructanase-treated *A*. *tequilana* stem juice (d).

The total moisture content of whole *Agave* leaves is upwards of 89% (Fig 2). Pressing released 69% of the fresh weight as a sugar-rich juice that was analyzed for glucose, fructose and sucrose content. The amounts of these directly fermentable sugars were also measured in *A*. *tequilana* stem juice, which is commonly used for tequila production. *A*. *americana* leaves and *A*. *tequilana* stems had similar amounts of free sugars in the juice (38–39 g/L), with a lower level detected in *A*. *tequilana* leaves (Fig 8a). Glucose was the most abundant sugar in all three samples although stem juice had a similar amount of sucrose. Additional, unidentified oligosaccharides were also detected in the raw juice samples (Fig 8b), indicating that these monosaccharide values were likely to be an underrepresentation of the total sugar content.

Two methods were used to hydrolyze the unidentified oligosaccharides into monosaccharides: 1) a non-specific acid hydrolysis using trifluoroacetic acid (TFA); and 2) specific enzymatic cleavage of fructans by a broad specificity fructanase. This fructanase exhibits both *exo*-inulinase activity, which degrades sucrose and kestose (glucose-fructose-fructose), and *endo*-inulinase activity, which liberates fructose from the non-reducing ends of long-chain fructans. Both TFA (Fig 8c) and fructanase (Fig 8d) cleaved the unidentified oligosaccharides completely into glucose and fructose, confirming that these oligosaccharides were fructans.

The total concentration of fermentable hexose sugars after hydrolysis in leaf samples was 41–48 g/L and increased to 104 g/L in *A*. *tequilana* stem juice. Fructose accounted for 68% of the stem monosaccharides, comparable to previous studies that found 60% of the total soluble sugars in *A*. *tequilana* stem to be fructans [10]. Galactose and galacturonic acid were detected in hydrolyzed juice samples at less than 0.5 g/L.

Inorganic elements in leaf juice that may affect fermentation were measured and compared with the inorganic content of whole leaf (S2 Table). The concentration of inorganic elements in *A*. *tequilana* juice was twice as high as in *A*. *americana* juice, although whole *A*. *americana* leaves had 20% more inorganic elements than *A*. *tequilana* leaves. High levels of calcium were observed in both species, particularly *A*. *americana* whole leaves, which may be attributed to inorganic calcium oxalate crystals detected in the tissue (Fig 5). Calcium levels in *A*. *tequilana* juice and whole leaves were similar to each other, but much higher than *A*. *americana* juice and much lower than *A*. *americana* whole leaf. It is possible that the difference in calcium detected between the two *Agave* species is an artefact of the shredding processes or different growing conditions for the two species.

#### *Agave* fibers are predominantly crystalline cellulose

With increasing reliance on synthetic fibers to meet consumer demands, production and markets for *Agave* fibers has been on the decline [14]. In recent years research has begun to investigate *Agave* fibers for emerging markets such as use in thermoplastics [63,64]. However, limited information is available regarding the composition of this waste material.

Crystalline cellulose comprised just under half (47–50% w/w) of the dry weight of fiber-enriched leaf fractions (Table 4), lower than the 68.4% w/w previously reported for crystalline cellulose in *A*. *americana* fibers [65]. The total cellulose in fibers of *A*. *lechuguilla* and *A*. *fourcroydes*, species specifically grown for their fibers, accounted for ~80% w/w of dry fiber weight, with the remainder composed mainly of lignin [66].

**Table 4:** Carbohydrates in fiber-enriched fractions from *Agave* leaves.

Component	*A*. *americana* (% w/w)	*A*. *tequilana* (% w/w)
Crystalline cellulose	47.2 ± 2.3	49.5 ± 1.9
Non-cellulosic polysaccharides	22.4 *± 0*.*8*	15.8 ± 1.3
* Arabinose*	*0*.*6 ± 0*.*1*	*0*.*3 ± 0*.*1*
* Glucose*	*8*.*6 ± 0*.*3*	*2*.*7 ± 0*.*6*
* Xylose*	*9*.*4 ± 0*.*9*	*11*.*4 ± 1*.*0*
* Other monosaccharides* *	*3*.*8 ± 0*.*1*	*1*.*4 ± 0*.*1*

Data are presented as a percentage of dry weight (% w/w).

*Includes mannose, rhamnose, glucuronic acid, galacturonic acid and galactose

Non-cellulosic polysaccharides accounted for 22.4% and 15.8% of the dry weight of *A*. *americana* and *A*. *tequilana* leaves, respectively. These values are consistent with the values reported in the literature suggesting that *A*. *tequilana* fibers contain 17% w/w non-cellulosic polysaccharides [67]. Xylose and glucose were the most abundant monosaccharides detected in the fibers after hydrolysis in 1M sulfuric acid, agreeing with linkage analysis that detected heteroxylans and xyloglucan in insoluble leaf fractions. In addition, similar to other studies [67] about ~30% of the fiber mass for both species was unaccounted for which may be attributed to unidentified or unhydrolyzed carbohydrates, lignin, inorganic compounds and protein.

### Fermentation of Agave juice

*A*. *tequilana* leaf juice was used as a substrate to investigate fermentation efficiency using two different strains of *Saccharomyces cerevisiae*. *A*. *tequilana* juice was autoclaved to minimize microbial contamination from native organisms and inoculated with one yeast strain. Sugar content of the starting juice was 41.4 g/L of total sugars and 30.0 g/L of readily fermentable WSC. After 96 h, both strains produced ethanol concentrations of 11–14 g/L (Table 5). Up to 90% of the monomers were fermented, which represent only 54–66% of the total sugars. Sugars in the *Agave* leaf juice, predominantly the fructans, are therefore being underutilized by these yeast strains.

**Table 5:** Fermentation of *Agave tequilana* leaf juice using *Saccharomyces cerevisiae*.

	Ethanol yield (96 hr)		
*S*. *cerevisiae* strain	Yield (g/L)	Conversion (% of total sugars)	Conversion (% of monomers)
139	11.4 ± 0.6	54%	74%
636	13.8 ± 0.5	66%	90%

Two strains of *S*. *cerevisiae* were used to ferment untreated *A*. *tequilana* leaf juice with a starting sugar concentration of 41.4 g/ L and WSC concentration of 30.0 g/L. Conversion efficiencies are based on a maximum conversion rate of sugar to ethanol of 51.1% w/w.

Historically, *Saccharomyces cerevisiae* is the most readily studied and utilized yeast for alcoholic fermentation assays [68] and can efficiently convert sucrose, glucose and fructose [69]; the main sugars in *Agave* leaf juice. However, alternative microorganisms may be more efficient at fermenting *Agave* juice sugars. For example, microorganisms such *Kluyveromyces marxianus* and *Torulaspora delbrueckii*, isolated from fermenting mezcal (a distilled alcohol made from *Agave*), express enzymes that hydrolyze fructooligosaccharides [70]. Activation of fructanase enzymes was induced by Ca^2+^, which is present in significant amounts in the leaves and juice of both *A*. *americana* and *A*. *tequilana* (S2 Table) [71]. In addition, using organisms such as *Escherichia coli* that can catabolise galacturonic acid may be a sensible choice for *Agave* if the pectic sugars in leaf tissue are to be fermented [72]. The use of readily studied *S*. *cerevisae* strains should thus be considered a benchmark by which to judge other organisms since it may be not be optimal for *Agave*. Careful selection of fermenting organisms may obviate the need for expensive pre-treatment processes or use of additional enzymes, which would increase the return on investment of using *Agave* spp. for biofuel production.

### *Agave* ethanol yields rival current biofuel feedstocks

Ethanol yields from three different *Agave* substrates were modelled: 1) the dry mass of the entire *Agave* plant based on leaf sugar composition, thereby underestimating sugar content because the additional sugar in the stem is not accounted for; 2) waste *A*. *tequilana* leaves from tequila production, and 3) juice from *A*. *tequilana* and *A*. *americana* leaves (Table 6). Theoretical ethanol yields were calculated using standard conversion assumptions [73].

**Table 6:** Theoretical ethanol yields for lignocellulosic feedstocks.

Biomass	Source of sugars	Ethanol yield (L/t)	Productivity (t/ha/yr)	Ethanol yield (L/ha/yr)
Corn	Stover without cobs	362−456*	3[18]	1086−1369
Wheat	Straw	406*	2.6[74]	1055
Sugarcane	Bagasse	318−500*	10[18]	3179−4996
Sorghum	Whole plant	268*	24−32.5[75,76]	6430−8708
Switchgrass	Whole plant	392−457*	5.2−23[77,78]	2036−10508
Poplar	Whole tree, no leaves	419−456*	5−11[18]	2096−5011
*Agave*	Whole residue	347*	10−34[18]	3474−11811
*A*. *americana*	Whole plant, extrapolated from leaf sugar content	437^^^	10−34[18]	4368−14851
*A*. *tequilana*	Whole plant, extrapolated from leaf sugar content	401^^^	10−34[18]	4009−13636
*A*. *tequilana* leaves	Whole leaf	401^^^	5.7−19^#^	2273−7728
*A*. *americana* leaves	Juice^†^	34^^^ ^,^ ^‡^	34−115.7^‡^	1165−3961
*A*. *tequilana* leaves	Juice^†^	30^^^ ^,^ ^‡^	23.4−79.7^‡^	691−2350

*Calculations were based on the compositional values listed in Table 1 [5].

^^^Calculations based on data obtained in this study.

^#^Assumes that 56.7% dry w/w of the whole 3 year old plants is leaf material [8].

^†^Assumes that juice accounted for 69% of plant wet weight; *A*. *americana* leaf was 88.5% w/w water; and *A*. *tequilana* leaf was 83.3% w/w water.

^‡^ Tonnes of wet weight rather than dry weight. Units for data are given in table headings. Constants for ethanol calculations are consistent with the National Renewable Energy Laboratory Theoretical Ethanol Yield Calculator [73]: 1.111 kg monomeric C6 sugar per 1 kg polymeric C6 polymer (glucan, fructan); 1.1363 kg monomeric C5 sugar per 1 kg polymeric C5 polymer (xylan, arabinan); 0.51 kg of ethanol produced from 1 kg of sugar. Productivity per hectare is based on previous studies [18,74–78].

The theoretical ethanol yield values for the whole leaf sugars of *A*. *americana* and *A*. *tequilana* were 437 L/t and 401 L/t, respectively. These values are comparable to estimates for other lignocellulosic biofuel feedstocks such as corn stover, sugarcane and switchgrass (Table 6). However, *Agave* plants may out-perform current biofuel feedstock crops in terms of productivity per hectare. Whole *A*. *tequilana* plants were predicted to yield 4000–13600 L/ha/yr and *A*. *americana* plants were predicted to yield 4400–14800 L/ha/yr. At the low end, these values exceed theoretical yields from first-generation feedstocks such as corn, wheat (*Triticum aestivum)* and sugarcane and at the high end, they double the yields of more recently investigated second generation feedstocks such as poplar, sorghum and switchgrass. The current values are consistent with those reported previously in the literature, which estimated that ethanol yields for *Agave* spp. may range from 3000–12000 L/ha/yr [18,20].

Waste *A*. *tequilana* leaves could generate 2300–7900 L/ha/yr and increase the value of existing *Agave* industries. However, since the majority of the mass of *Agave* plants is water, it may be more economically viable to directly separate and ferment the sugar-rich juice, which could yield 690–4000 L/ha/yr (Table 6). Even using a generic *S*. *cerevisiae* strain unadapted to *Agave* substrates, yields of up to 1500 L/ha/yr from *A*. *tequilana* leaf juice and 2600 L/ha/yr from *A*. *americana* leaf juice could be obtained (assuming a fermentation conversion of 66% for both substrates; Table 5). More efficient fermenting organisms may increase the value of using *Agave* juice as a biofuel feedstock in terms of yield and revenue returns.

It is worth noting that *Agave* cultivation systems have not yet been optimized to produce sugar for biofuel and biochemical industries. Information about agronomical practices, such as planting density or the optimal age to harvest the plants, is limited. If the plants are harvested at 2–3 years of age rather than the traditional 8–12 years of age, plant spacing could be reduced further, increasing density per hectare. In addition, further information about microorganisms that are naturally found within *Agave* may be beneficial for the industries that grow and commercialize these plants. In a biofuel context, it may be useful to isolate and characterise organisms that naturally grow on *Agave*, as they presumably utilize sugars such as fructans efficiently and are tolerant to a range of environmental conditions. The isolation and use of microorganisms found on or within biomass for the conversion of carbohydrates to biofuel is not novel; grape marc, an agro-industrial waste material, has been found to be a rich source of robust organisms that are economically and productively favourable for second generation bioethanol conversion [79]. Further research is required to identify the microorganisms associated with the *Agave* microbiome.

## Conclusion

The leaf tissues of *A*. *americana* and *A*. *tequilana* species contain 56–60% (dry weight) of potentially fermentable sugars, over half of which are present in a soluble fraction. These same tissues also contain relatively low amounts of lignin. Ethanol yields (ha/yr) that could be generated from *Agave* leaves and whole plants rival those of the most successful biofuel feedstock crops such as switchgrass and poplar. *Agave* differs from most common feedstocks in its high moisture content, but nearly 70% of plant mass can be extracted with simple mechanical pressing to release a sugar-rich juice. Crushing and fermenting the juice on site without any pre-treatment can produce competitive ethanol yields, with room for improvement by judicious selection of fermenting organisms, and by-products may be produced from the crystalline cellulose enriched bagasse waste. The comprehensive compositional data for *Agave* leaves and fermentation trials reported herein will be instrumental in the development of agronomic, saccharification and fermentation methods for converting *Agave* raw material into biofuel or biochemical products.

## Supporting Information

S1 Table Monosaccharide linkage analysis data for *Agave* leaves (mol%). Analysis completed on alcohol insoluble residues (AIR). Data are presented as relative percent molarity (mol%).
(DOCX)

S2 Table Elemental analysis of *Agave* juice and whole leaf. Data are presented as mg/kg of material. ^1^Average of two biological replicates. ^2^Average of three biological replicates.
(DOCX)
