# Supplementary material for: Prospecting for Energy-Rich Renewable Raw Materials: Agave Leaf Case Study
Source: PLoS One. 2015 Aug 25;10(8):e0135382. doi: 10.1371/journal.pone.0135382 (PMC4549257; doi:10.1371/journal.pone.0135382)
Supplement: S1 Table — Analysis completed on alcohol insoluble residues (AIR). Data are presented as relative percent molarity (mol%). (DOCX) [file pone.0135382.s001.docx]

**Supporting Information Table 1**

**Monosaccharide linkage analysis data for *Agave* leaves (mol%)**

| **Polysaccharide** | **Derivative linkage** | ***A. americana* (mol%)** | ***A. tequilana* (mol%)** |
| --- | --- | --- | --- |
| Arabinan | *1,5 - Ara (f)*  *1,2,5- Ara (f)* | 4.8 ± 0.8  0.7 ± 0.2 | 4.4 ± 0.2  0.3 ± 0.2 |
| Type I arabinogalactan | *1,4-Gal (p)*  *1,4,6-Gal (p)* | 6.5 ± 2.1  0.9 ± 0.5 | 2.3 ± 0.7  0.0 |
| Type II arabinogalactan | *1,6-Gal (p)*  *1,3,6-Gal (p)*  *t-Gal* | 0.2 ± 0.1  1.1 ± 0.4  1.1 ± 0.2 | 0.2 ± 0.0  0.7 ± 0.3  0.7 ± 0.2 |
| Glucuronarabinoxylan | *1,4-Xyl (p)*  *1,2,4-Xyl (p)*  *1,3,4-Xyl (p)*  *1,2,3,4-Xyl (p)*  *t-Ara* | 9.9 ± 2.3  0.6 ± 0.2  0.2 ± 0.1  0.6 ± 0.4  2.1 ± 1.2 | 11.6 ± 2.0  1.1 ± 0.2  0.5 ± 0.2  1.2 ± 0.6  1.9 ± 0.2 |
| Cellulose | *1,4-Glc(p)* | 31.9 ± 2.1 | 45.3 ± 4.2 |
| Heteromannan | *1,4-Man (p)*  *1,4-Glc (p)* | 3.3 ± 0.3  3.3 ± 0.4 | 3.0 ± 0.3  3.0 ± 0.3 |
| Homogalacturonan | *1,4-Gal A (p)*  *t-Gal A (p)* | 16.8 ± 1.1  0.9 ± 0.4 | 5.9 ± 1.9  0.6 ± 0.1 |
| Rhamnogalactan I/II | *1,2,4-Rha (p)* | 0.7 ± 0.3 | 0.3 ± 0.0 |
| Xyloglucan | *1,4,6-Glc (p)*  *1,4-Glc (p)*  *1,2-Xyl (p*  *1,2-Gal (p)*  *t-Fuc (p)*  *t-Xyl (p)* | 3.1 ± 0.3  3.1 ± 2.1  1.1 ± 0.3  0.7 ± 0.1  0.7 ± 0.5  2.0 ± 0.6 | 3.7 ± 0.4  3.7 ± 4.2  1.3 ± 0.2  0.6 ± 0.1  0.9 ± 0.3  2.6 ± 0.2 |

Analysis completed on alcohol insoluble residues (AIR). Data are presented as relative percent molarity (mol%).
