# Supplementary material for: Prospecting for Energy-Rich Renewable Raw Materials: Agave Leaf Case Study
Source: PLoS One. 2015 Aug 25;10(8):e0135382. doi: 10.1371/journal.pone.0135382 (PMC4549257; doi:10.1371/journal.pone.0135382)
Supplement: S2 Table — Data are presented as mg/kg of material. 1Average of two biological replicates. 2Average of three biological replicates. (DOCX) [file pone.0135382.s002.docx]

**Supporting Information Table 2**

**Elemental analysis of *Agave* juice and whole leaf**

| **Element** | **Whole leaf** | | **Juice** | |
| --- | --- | --- | --- | --- |
|  | *A. americana*^2^  (mg/kg) | *A. tequilana*^2^  (mg/kg) | *A. americana*^2^  (mg/kg) | *A. tequilana*^1^  (mg/kg) |
| Aluminium (Al) | 5 | 24 | 0 | 1 |
| Calcium (Ca) | 16733 | 3400 | 873 | 3800 |
| Iron (Fe) | 36 | 70 | 1 | 47 |
| Magnesium (Mg) | 10667 | 10007 | 883 | 1190 |
| Phosphorus (P) | 1073 | 4767 | 54 | 720 |
| Potassium (K) | 20700 | 20400 | 2033 | 2500 |
| Sodium (Na) | 20 | 81 | 4 | 17 |
| Sulfur (S) | 657 | 693 | 42 | 72 |
| Zinc (Zn) | 18 | 21 | 4 | 6 |
| **Total** | **49909** | **39463** | **3895** | **8353** |

Data are presented as mg/kg of material. ^1^Average of two biological replicates. ^2^Average of three biological replicates.
